# Interactions between Distant ceRNAs in Regulatory Networks

**DOI:** 10.1016/j.bpj.2014.03.040

**Published:** 2014-05-20

**Authors:** Mor Nitzan, Avital Steiman-Shimony, Yael Altuvia, Ofer Biham, Hanah Margalit

**Affiliations:** † Racah Institute of Physics, The Hebrew University of Jerusalem, Jerusalem, Israel; ‡ Department of Microbiology and Molecular Genetics, Institute for Medical Research Israel-Canada, Faculty of Medicine, The Hebrew University of Jerusalem, Jerusalem, Israel

## Abstract

Competing endogenous RNAs (ceRNAs) were recently introduced as RNA transcripts that affect each other’s expression level through competition for their microRNA (miRNA) coregulators. This stems from the bidirectional effects between miRNAs and their target RNAs, where a change in the expression level of one target affects the level of the miRNA regulator, which in turn affects the level of other targets. By the same logic, miRNAs that share targets compete over binding to their common targets and therefore also exhibit ceRNA-like behavior. Taken together, perturbation effects could propagate in the posttranscriptional regulatory network through a path of coregulated targets and miRNAs that share targets, suggesting the existence of distant ceRNAs. Here we study the prevalence of distant ceRNAs and their effect in cellular networks. Analyzing the network of miRNA-target interactions deciphered experimentally in HEK293 cells, we show that it is a dense, intertwined network, suggesting that many nodes can act as distant ceRNAs of one another. Indeed, using gene expression data from a perturbation experiment, we demonstrate small, yet statistically significant, changes in gene expression caused by distant ceRNAs in that network. We further characterize the magnitude of the propagated perturbation effect and the parameters affecting it by mathematical modeling and simulations. Our results show that the magnitude of the effect depends on the generation and degradation rates of involved miRNAs and targets, their interaction rates, the distance between the ceRNAs and the topology of the network. Although demonstrated for a miRNA-mRNA regulatory network, our results offer what to our knowledge is a new view on various posttranscriptional cellular networks, expanding the concept of ceRNAs and implying possible distant cross talk within the network, with consequences for the interpretation of indirect effects of gene perturbation.

## Introduction

**Figure 1:**
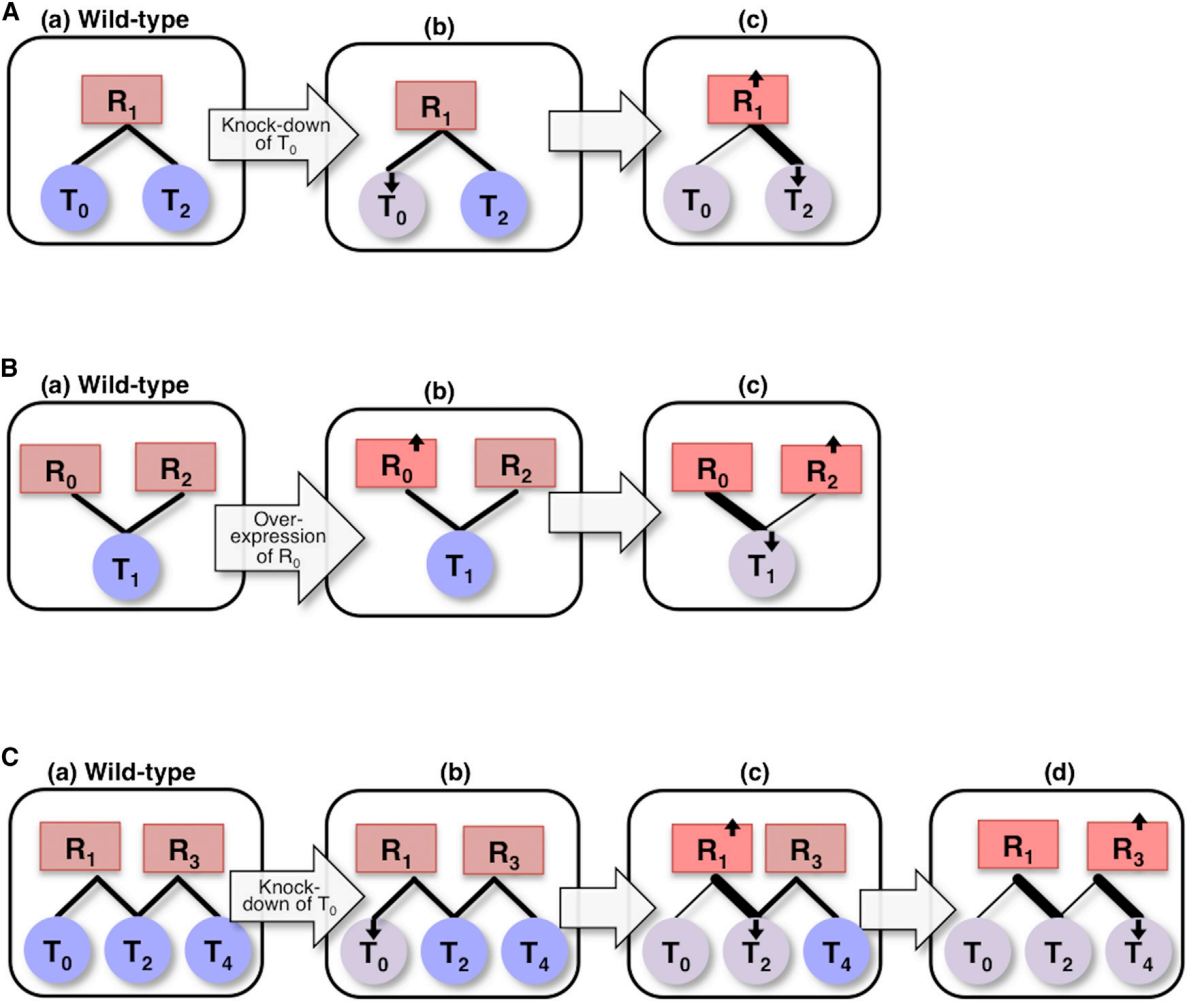
Propagation of perturbation effects through coregulation and target sharing. (*A*) Mutual effects between mRNAs competing for binding the same miRNA regulator (competing endogenous mRNAs). R_1_ is a miRNA that negatively regulates its targets T_0_ and T_2_ (*A a*). Upon knockdown of T_0_ (*A b*), more R_1_ molecules are freed (*A c*). The freed R_1_ molecules interact with T_2_, exerting greater regulatory effect on T_2_. As a result, the level of free T_2_ molecules is further decreased (*A c*). Thus, the change in T_0_ level affects T_2_ level through their common regulator. Rectangles correspond to miRNA regulators and circles correspond to target mRNAs. The intensity of node color and the direction of the arrow within the node mark the direction of change in expression. The thickness of the edge marks the strength of the regulation. (*B*) Mutual effects between miRNAs over binding to a shared target (competing endogenous miRNAs). R_0_ and R_2_ are two miRNAs that negatively regulate T_1_ (*B a*). Overexpression of R_0_ (*B b*) leads to downregulation of T_1_ (*B c*). As a result, the level of free R_2_ molecules increases (*B c*). (*C*) Propagation of perturbation effect. R_1_ and R_3_ are two miRNAs that negatively regulate (T_0_, T_2_) and (T_2_, T_4_), respectively (*C a*). T_2_ is a shared target of R_1_ and R_3_. Knockdown of T_0_ (*C b*) increases the level of free R_1_ molecules (*C c*). The freed R_1_ molecules bind T_2_, further downregulating T_2_ (*C c*). The decrease in T_2_ level frees more R_3_ molecules to focus on T_4_, which, in turn, is further downregulated (*C d*). In such a case, there is a propagation of the effect of T_0_ knockdown to T_4_ through T_2_, the shared target of R_1_ and R_3_. Note that in rows *A*–*C*, the index of the perturbed node (source) is 0 and regulator and target nodes are not indexed separately but interchangeably, by their distance from the source.

MicroRNAs (miRNAs) play major roles in posttranscriptional regulation of gene expression. This family of molecules comprises ∼22-nucleotide-long RNAs that mostly function as negative regulators of protein expression. They exert their regulatory function by basepairing with the RNA transcripts of their targets when bound to the Argonaute (AGO) proteins, inhibiting translation and/or destabilizing the RNA transcript (1). It was shown that various RNA transcripts, such as lincRNAs, circRNAs, pseudogenes and even mRNAs, may affect each other’s expression level through competition for common miRNA regulators (e.g., (2–12); for review, see Tay et al. (13)). RNA transcripts that are coregulated by a miRNA and exhibit such mutual effects were termed competing endogenous RNAs (ceRNAs) (14). It was demonstrated experimentally that decreasing or increasing the expression level of a ceRNA leads to a respective decrease or increase in other coregulated ceRNAs (10,11,15). The response of ceRNAs to perturbations in coregulated ceRNAs was also characterized computationally (11,16,17). ceRNAs were shown to play a role in critical cellular pathways. For example, in *Arabidopsis thaliana*, it was shown that under phosphate starvation, a mimic of miR-399 target was highly expressed and attracted most of the miR-399 molecules, relieving the repression of its target PHO2, which is needed in relatively high levels under this biological condition (2). Another example regards the tumor suppressor PTEN, whose level decreases in some cancer cells. It was shown that one of the mechanisms leading to this decrease involves the depletion or deletion of ceRNAs of PTEN (e.g., the transcript of the pseudogene PTENP1) (4,10,15,18,19). The depleted ceRNAs freed the common miRNA regulators to further bind PTEN and decrease its level. This implies bidirectional effects between miRNAs and their target RNAs, where a change in the expression level of one target affects the level of the miRNA regulator, which in turn affects the level of other targets (Fig. 1 *A*).

Recently, ceRNAs were examined in the context of other cellular networks, and were shown to be tightly integrated within the transcriptional regulatory network (11). In general, application of network analysis tools to biological large-scale data has introduced a novel view to gene functionality and has provided many new insights into the structure of molecular pathways (e.g., (20–24)). Although numerous such analyses were applied to the transcriptional regulatory network, analysis of the posttranscriptional regulatory network (PTRN) of miRNA-target interactions was much more limited. This stems from two main difficulties that we overcome in this work. 1), Although there are ample experimental data about transcriptional regulatory interactions that can be analyzed, until recently such reliable high-throughput data were lacking for the PTRN. A recent source of experimental miRNA-target interactions, provided by the cross-linking, ligation, and sequencing of hybrids (CLASH) methodology (25), makes it possible for the first time to construct a large-scale experimentally determined network of direct interactions between miRNAs and their target RNAs within a cell. 2), The high connectivity of the transcription regulation network (due to chained transcription regulation interactions) has enabled extensive analyses of paths in the network and the identification of indirect and long-range interactions (e.g., (21–23,26,27)). In contrast, miRNAs are not known to extensively bind each other, and therefore, the depth of connectivity and length of possible paths that could be followed in the directed network of miRNA-target interactions were very limited. However, we suggest that due to the bidirectional effects between miRNAs and targets, the miRNA-mRNA network should be better represented as an undirected graph rather than by the conventional directed graph representation, as was indeed depicted in several recent works (e.g., (16,17)). This provides a different view of the PTRN connectivity, which allows tracing paths and information flow in this network (Fig. 1).

Here, we analyze the undirected network of miRNA-target interactions deciphered experimentally in HEK293 cells. We demonstrate that it is a dense network, implying short paths between most nodes. This suggests the occurrence of many ceRNAs that are not coregulated by the same miRNA but appear along a path of alternating coregulated target mRNAs and miRNAs with shared targets (Fig. 1), implying that the effect of gene perturbation may propagate and affect the expression levels of distant genes, as noted also by Bosia et al (17). We study this type of information flow between distant ceRNAs in the network by mathematical modeling and simulation, as well as by analysis of experimental perturbation data. Cross talk between distant ceRNAs may provide possible explanations for many previously inexplicable results, including indirect effects of gene perturbations, off-target effects of miRNAs, and side effects of drugs directed at specific genes.

## Materials and Methods

### Mathematical model

The model we present below describes the change over time of the levels of miRNAs, their target RNAs, and their complexes. The wiring diagrams and dynamical processes of the miRNA-target RNA interactions were translated into a set of coupled ordinary differential equations under the assumption of mass-action kinetics for all reactions. The equations were implemented in MATLAB (MathWorks, Natick, MA) and integrated using its built-in solver ode45.

### Determination of the miRNA-mRNA interactome and network analysis

The human miRNA-mRNA interactome we analyzed is based on results using the CLASH methodology (25), a technique that ligates and sequences miRNA-target RNA duplexes associated with human AGO1. The CLASH methodology was applied to HEK293 cells and identified miRNA-mRNA interactions in the 3′UTR, as well as in the 5′UTR and CDS of the targets. In addition, miRNA interactions were found with other noncoding RNAs, such as tRNA and lincRNA. Despite the wide scope of interactions identified, it should be noted that the miRNA interactome identified by the CLASH method likely samples only part of the complete miRNA interactome.

For consistency with the miR-92a microarray data (see below), which used Human Exon Array, we considered only miRNA-mRNA interactions for all analyses in this study. Although the CLASH interactome lists the reads of individual mRNA transcripts, for this analysis we summed up the reads of all mRNA transcripts of the same gene. Since the results using miRNA-mRNA interactions filtered for read number (>5) were consistent with the results using all reads, we did not filter for read number in our analysis. Unless otherwise specified, in all analyses we used a set of high-confidence interactions, as determined by Helwak et al. (25), i.e., miRNA-mRNA interactions with binding free energy <−13.4 kcal/mole.

Network figures were created using Cytoscape (28), and the average clustering coefficient (C) and average shortest path length (ASPL) were analyzed using the Cytoscape Network Analyzer Plugin (29) and the Python NetworkX package (30). The small world coefficient (*σ*) for the two bipartite projections was calculated according to the equation *σ* = (C/C_rand_)/(ASPL/ASPL_rand_), where X_rand_ refers to a property averaged over instantiations of random Erdős-Rényi networks. *σ* > 1 indicates a small world network (31). One hundred random Erdős-Rényi networks with number of nodes and edge probability equivalent to the bipartite projections were created using NetworkX. C and ASPL of these random networks were measured and averaged to calculate *σ*.

### Microarray data and perturbation analysis

CEL files of the miR-92a depletion microarray experiment (25) were downloaded from GEO (GSE46039) and analyzed using PARTEK and the MATLAB Bioinformatics and Statistical Toolboxes. Log2 fold-change values from the microarray data were normalized to set the median of the total microarray data to 0. miR-92a is an abundant miRNA in HEK293, and it was depleted by transfection of the anti-miR-92a oligonucleotides (here termed source), which compete with miR-92a natural targets and thus reduce its endogenous activity. To study the propagation of perturbation effect, genes from the microarray study were divided into groups: miR-92a targets separated by two edges from the perturbed source (2-sep targets), targets separated by four edges from the source (4-sep targets), targets separated by six edges from the source (6-sep targets), and control genes, as described in Results. Note that in defining the groups we used all CLASH data including low-confidence targets to avoid the assignment of potential direct targets as distant ceRNAs. The perturbation analysis was applied once to all the reported CLASH interactions and once to the high-confidence interactions. For all reported CLASH interactions, the size of the groups was larger (2-sep targets, n = 168; 4-sep targets, n = 4905; and 6-sep targets, n = 32) than for the high-confidence interactions (2-sep targets, n = 150; 4-sep targets, n = 4545; and 6-sep targets, n = 25). It should be noted that the microarray data was missing some of the mRNA targets from the CLASH data. Throughout the analysis, a two-sided Kolmogorov-Smirnov test (K-S test) was used in comparing the log2 fold-change values between groups, as mentioned in Results. The analysis of transcription factor effects, classification of activators and repressors, as well as their targets, was based on the Bioknowledge database (www.biobase-international.com/transcription-factor-binding-sites). The definition of a control group of highly expressed genes in HEK293 cells was based on the Human Protein Atlas (32).

## Results

### Modeling miRNA-target RNA interaction

We first demonstrate by mathematical formulation the bidirectional effects between a miRNA and its target RNA. We described the temporal variation in the levels of a miRNA and its target RNA by rate equations, similarly to previous models (11,16,17), where the miRNA is a negative regulator and the miRNA and target form a complex by diffusion-mediated interaction, rendering the target RNA not functional, either by destabilization or by inhibited translation (in the case of mRNA targets). For simplicity, we refer to miRNA-target interaction resulting in destabilization, manifested as degradation, as elaborated below. In addition, it is assumed that all miRNAs are bound to AGO and functional.

(1) $$\begin{array}{l} \frac{dR}{dt}=g_{R}-b\;R\;T+u_{C}\;C+\left(1-\alpha \right)d_{C}\;C-d_{R}\;R\\ \frac{dT}{dt}=g_{T}-b\;R\;T+u_{C}\;C-d_{T}\;T\\ \frac{dC}{dt}=b\;R\;T-u_{C}\;C-d_{C}\;C. \end{array}$$

In these equations, *R* represents the level of the miRNA regulator free molecules (those not bound to the target), *T* represents the level of free target RNA molecules, and *C* represents the level of the miRNA-target complexes. Note that we use roman letters when referring to molecular entities (e.g., T_0_ in Fig. 1) and italic letters when referring to the levels of these molecular entities (e.g., *T* in Eq. 1). The parameters *b* and *u*_*C*_ represent the miRNA-target binding and unbinding rates, respectively. *g*_*R*_, *d*_*R*_, and *g*_*T*_, *d*_*T*_, represent the generation and degradation rates of the miRNA and target RNA, respectively. *d*_*C*_ represents the degradation rate of the miRNA-target complex. The parameter *α* (0 ≤ *α* ≤ 1), termed the stoichiometric factor, represents the measure of the stoichiometricity of the miRNA action on its target, ranging between the catalytic (*α* = 0) and the stoichiometric (*α* = 1) case. For the catalytic case, a single miRNA molecule can bind and lead to the sequential degradation of several target RNA molecules, whereas for the stoichiometric case, both miRNA and target RNA are degraded when bound in a complex. The steady-state solutions,

(2) $$T=\frac{g_{T}}{d_{T}+b\;R\left(1-\frac{u_{C}}{u_{C}+d_{C}}\right)}\;R=\frac{g_{R}}{d_{R}+b\;T\left(1-\frac{u_{C}+\left(1-\alpha \right)d_{C}}{u_{C}+d_{C}}\right)}$$

demonstrate the bidirectional effects between regulator and target, where the target RNA level increases as the miRNA level decreases and vice versa. The recognition of these bidirectional effects between miRNA and target implies that the PTRN can be treated as an undirected network, enabling the analysis of pathways and information flow, which was inherently limited when relating to the PTRN as directed.

### The human miRNA-mRNA interactome is a highly connected undirected network

**Figure 2:**
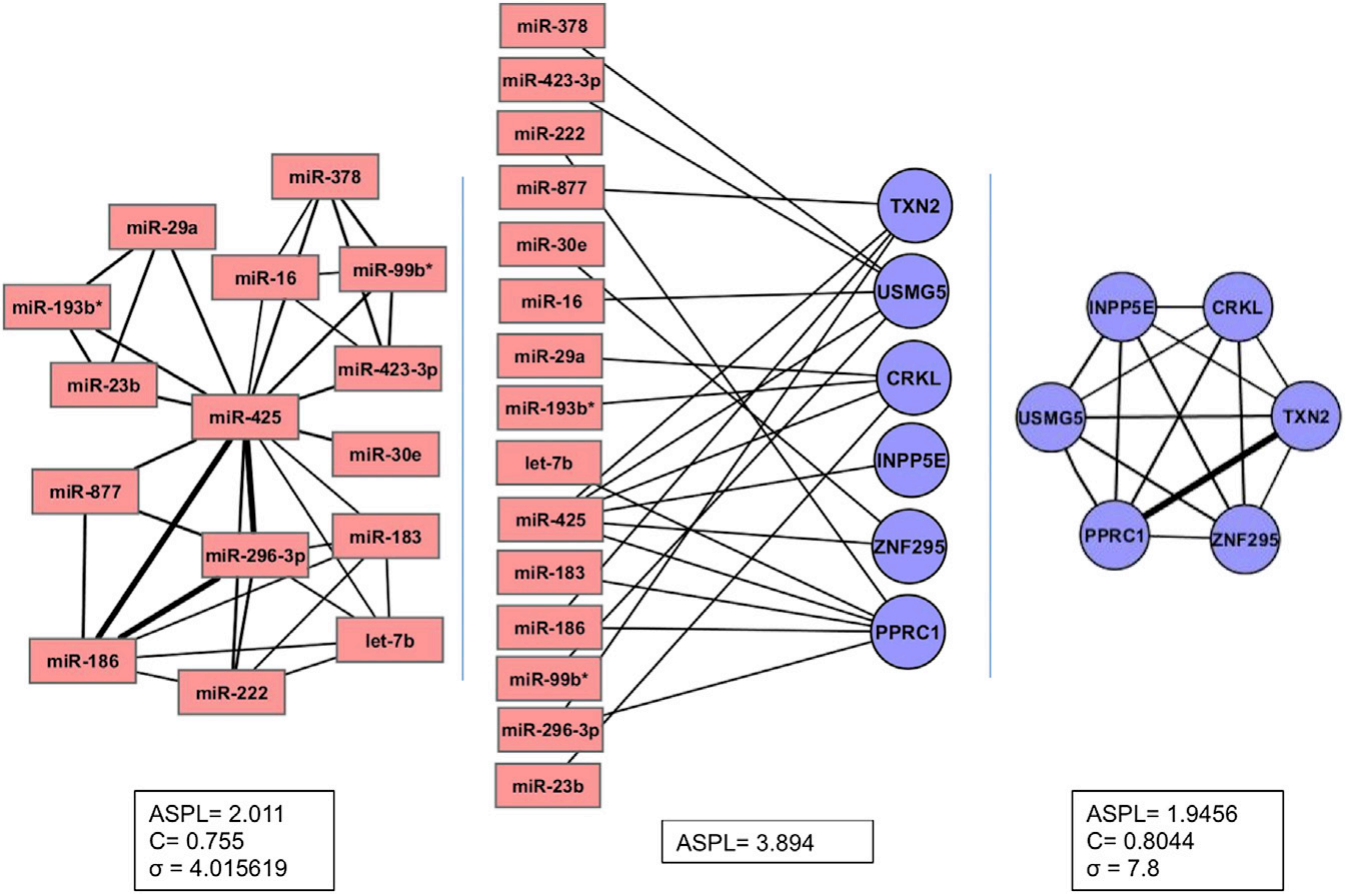
A representative subnetwork of the human miRNA-mRNA bipartite network. (*Center*) A schematic representation of the network based on a sample of the CLASH data (25). Rectangles correspond to miRNA regulators and circles correspond to target mRNAs. An edge is placed between a miRNA and a mRNA if the miRNA was shown to regulate the mRNA in the CLASH high-confidence data. (*Left*) The miRNA-miRNA projection of the schematic interactome bipartite network, in which two miRNAs are connected by an edge if they share a target. The edge width corresponds to the number of targets shared by the miRNAs. (*Right*) The mRNA-mRNA projection of the schematic interactome bipartite network, in which two mRNAs are connected by an edge if they are both regulated by the same miRNA. The edge width corresponds to the number of common miRNA regulators. For each network, the ASPL is specified; included as well are the average clustering coefficient, C, and small world coefficient, *σ*, which by definition are relevant only for the projected networks. The reported values for the various measures were computed for all the data and not only for the subnetwork presented here. To see this figure in color, go online.

To analyze the extent of connectivity within an experimental miRNA-target RNA network treated as an undirected network, we examined the human interactome of miRNAs and their targets in HEK293 cells, identified by the CLASH methodology (25). In this analysis, for technical consistency, we included only mRNA targets, which comprise the majority of targets (Materials and Methods). The network included 379 miRNAs and 6323 highly confident mRNA targets, represented as nodes, and 14,807 miRNA-mRNA interactions, represented as undirected edges (see Materials and Methods). A representative subnetwork is shown in Fig. 2 (*center*). Our analysis showed that the network is a scale-free network, as the node degree distribution highly correlates with a power law distribution (power law coefficient = 2.57; r = 0.95). The shortest path distribution was found to be sharply concentrated around the value of 4 (ASPL = 3.894; Fig. S1 in the Supporting Material). Thus, the miRNA-mRNA interactome is an extremely interconnected network. This network is in fact a bipartite graph, composed of two sets of nodes, miRNAs and mRNAs, where edges exist between, but not within, the two sets (Fig. 2, *center*). To better characterize the two sets, we examined two networks that represent complementary projections of the interactome. In the first network (Fig. 2, *right*), the nodes represent mRNAs, and edges connect mRNAs that are regulated by the same miRNA. In the second network (Fig. 2, *left*), the nodes represent miRNAs and edges connect miRNAs that share targets. The mRNA network is a small world network (small world coefficient, *σ* = 7.8; see Materials and Methods) that contains 6299 nodes and 1,997,581 edges, exemplifying the high connectivity between nodes (average clustering coefficient, C = 0.8044). In a similar way, the miRNA network is a small world network containing 355 nodes and 10,794 edges (C = 0.755, *σ* = 4.0). In correspondence with the miRNA-mRNA network, the majority of node pairs in both projected networks have shortest paths of length 2 (Fig. S1), indicating that they are separated by a single node. Overall, the highly connected nature of the miRNA-mRNA interactome implies the potential for information flow between many pairs of nodes through many pathways in the network.

### Characterizing information flow

**Figure 3:**
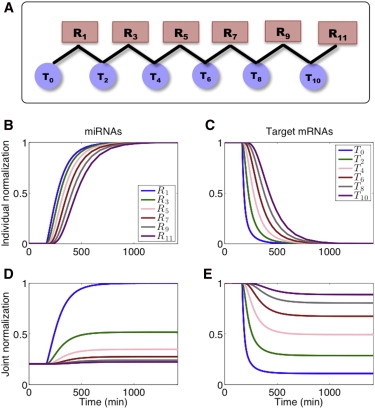
Simulation results of the propagation of a perturbation effect along a chain of miRNA-target interactions. (*A*) Schematic representation of the chain of interactions comprising recurrent interacting miRNAs and targets, denoted by R_*ℓ*_ and T_*ℓ*_, respectively. (*B–E*) The network in *A* is perturbed out of steady state by decreasing the generation rate of one of the targets in the chain, T_0_. Shown are the changes in miRNA levels (*B* and *D*) and target RNA levels (*C* and *E*) over time. (*B* and *C*) Levels are normalized by the maximal and minimal levels within each individual RNA component. (*D* and *E*) Levels are normalized by the maximal level of R_1_ (*D*) or T_0_ (*E*). A gradual increase and decrease in the levels of regulator miRNAs and target RNAs, respectively, is clearly observed, consistent with their distance from the perturbed source node, T_0_. Parameter values used in the simulations are reported in Table S1.

Recognizing the potential for information flow in the experimentally determined miRNA-mRNA network, we turned to characterize its properties and evaluate its significance. We expanded the basic model presented above and followed the effect of gene expression perturbation through a network of interacting miRNAs and targets (Fig. 2 and Fig. 3 *A*). As a first step toward understanding a complex network of interactions, we analyzed a chain model (Fig. 3 *A*). Although the chain model is a simplified representation of a real miRNA-mRNA network, lacking much of its complexity, it is able to capture the cross talk between distant ceRNAs and propagation of perturbation effects along pathways of the PTRN. In addition, we show that it is analytically tractable, thus allowing us to characterize the magnitude of cross talk between genes as a function of parameters and distance from the perturbed node.

Consider a chain of 2*N* interacting RNA components: *N* miRNAs and their *N* target mRNAs, so that every miRNA regulates two target mRNAs and each target mRNA is a shared target of two miRNAs (except for the RNA components at the boundaries). *ℓ* is an index representing the position of the RNA component along the chain relative to a source component (e.g., T_0_ in Fig. 3 *A*). *C*_*ℓ*,*ℓ*±1_ represents the level of the complex composed of the *ℓ*^th^ miRNA and (*ℓ* ± 1)^th^ target mRNA. We model this system by a set of rate equations, following the notations presented above:

(3) $$\begin{array}{ll} \frac{dR_{\ell }}{dt}=g_{R}-b\;R_{\ell }\left(T_{\ell -1}+T_{\ell +1}\right)+u_{C}\left(C_{\ell ,\ell -1}+C_{\ell ,\ell +1}\right)+\left(1-\alpha \right)d_{C}\left(C_{\ell ,\ell -1}+C_{\ell ,\ell +1}\right)-d_{R}R_{\ell } & \ell =1,\;3,\ldots ,2N-1\\ \frac{dT_{\ell }}{dt}=g_{T}-b\;T_{\ell }\left(R_{\ell -1}+R_{\ell +1}\right)+u_{C}\left(C_{\ell -1,\ell }+C_{\ell +1,\ell }\right)-d_{T}T_{\ell } & \ell =0,\;2,\ldots ,2N-2\\ \frac{dC_{\ell ,\ell \pm 1}}{dt}=b\;R_{\ell }T_{\ell \pm 1}-u_{C}C_{\ell ,\ell \pm 1}-d_{C}C_{\ell ,\ell \pm 1} & \ell =1,\;3,\ldots ,2N-1, \end{array}$$

**Figure 4:**
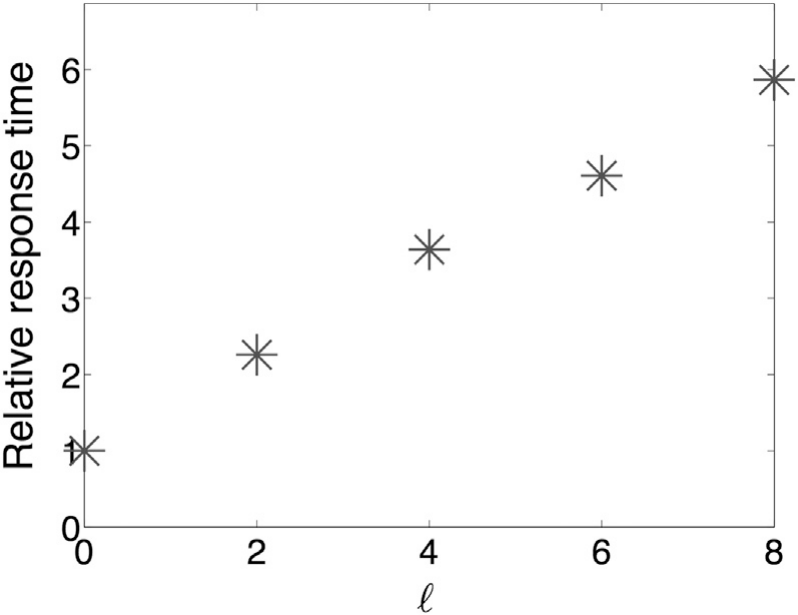
Relative response time versus distance. The response time, the time required for an RNA component to reach halfway to its new steady-state level following the perturbation of the source, is presented, normalized by the response time of the source. The relative response time increases with the distance of the RNA component from the source. Parameter values used in the simulations are reported in Table S1.

For clarity, we first assigned the same parameter values within each set of RNA components, as well as the same binding constants to all miRNA-target RNA pairs (parameter values are reported in Table S1). We consider the case where at time t = 0 the system is in steady state. Using the expanded model, it is possible to quantitatively follow the temporal variation in expression levels of miRNAs and target RNAs upon perturbation out of steady state of any component of the chain presented in Fig. 3 *A* (overexpression or underexpression of either regulator or target). The RNA components presented in Fig. 3 *A* are embedded within a longer chain, which yields an approximate shared steady state for the different miRNAs, as well as a shared steady state for the different target mRNAs. In general, the perturbed RNA component was classified as the source. For simplicity, we present the results for a subnetwork comprising a chain of 11 RNA components originating from the source. However, the perturbed source can be located anywhere within the chain, implying information flow in both directions. In the example demonstrated in Fig. 3, we present the effect of a decrease in the generation rate of T_0_ on the levels of the other RNA components over time. Such a perturbation results in a lower level of T_0_, which leads to an increase in the miRNA levels and a decrease in the levels of the other target RNAs in a successive manner (Fig. 3, *B*–*E*). The effect of the perturbation is delayed and decreases as the distance of an RNA component from the source increases. The response time, the time required for an RNA component to reach halfway to its new steady state following the perturbation of the source, increases with the distance of the RNA component from the source (Fig. 4).

To characterize the extent of information flow, we examined how the distance of an RNA component from the source affects the asymptotic change in its level. For concreteness, we focused here on the target RNAs. Similar analysis can be done for the miRNAs. To this end, we computed the correlation function, *C*(*ℓ*), defined as the normalized full derivative of the level of T_*ℓ*_ to the level of the source, T_0_,

(4) $$C\left(\ell \right)=\frac{dT_{\ell }/T_{\ell }}{dT_{0}/T_{0}},\;\ell =0,\;2,\;4,\ldots$$

**Figure 5:**
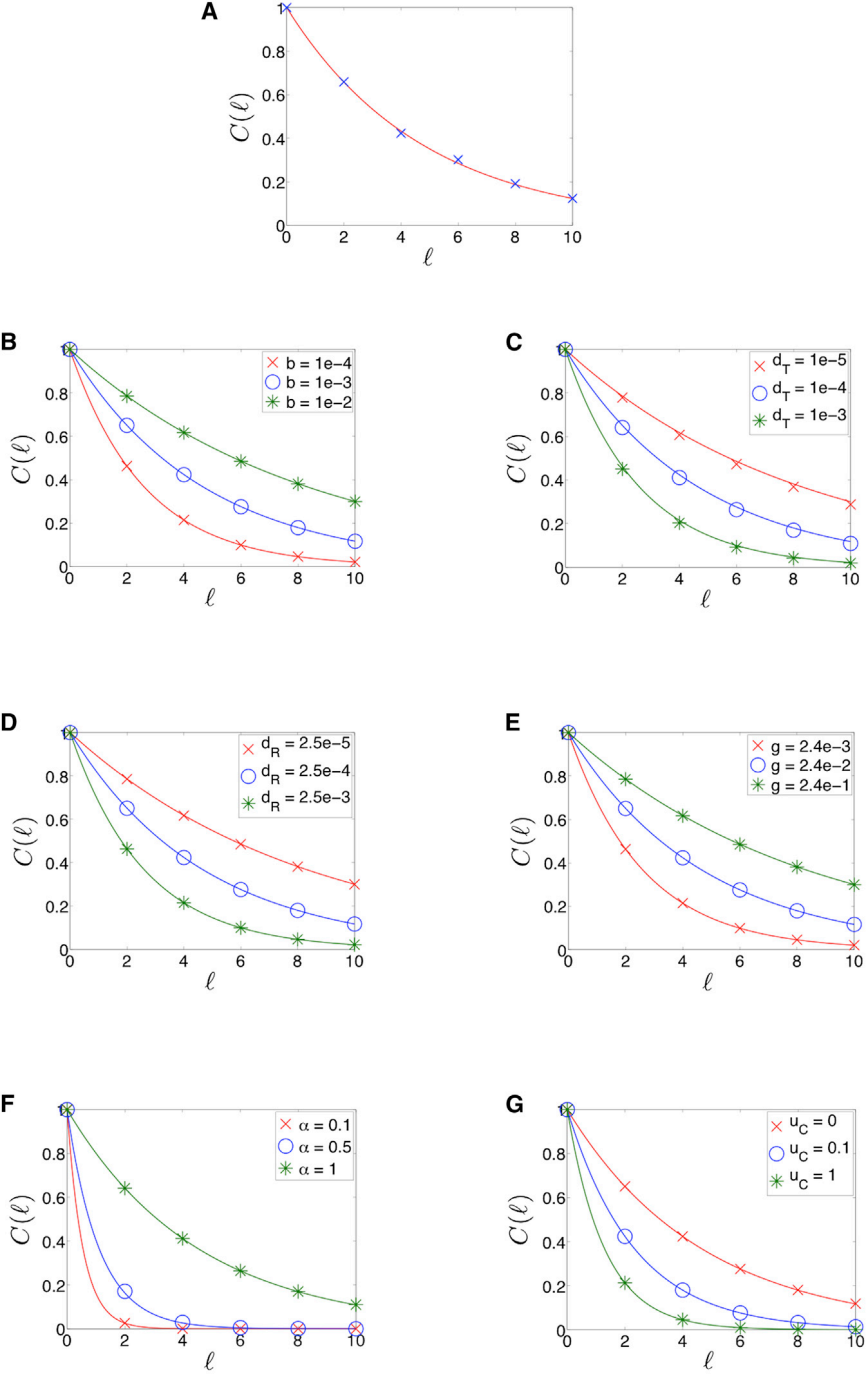
The correlation function decays in a near-exponential form as a function of the distance from the source. (*A*) Shown are results for a miRNA-target RNA chain in which the parameters are sampled from a suitable Gaussian distribution, located within the biologically relevant range. Parameter values used are reported in Table S1, with respective standard deviations that are each equal to 1/10 of the respective parameter value. The symbols represent the average value of the correlation function, *C*(*ℓ*), over 50 instantiations of the parameter values. The curve represents the exponential fit to the data, where *ℓ*_0_ = 2.181. (*B–G*) Symbols represent numerical results and lines represent analytical results. The value of *C*(*ℓ*) for RNA components along the chain increases as the miRNA-target binding rate increases (*B*), target RNA (*C*) and miRNA (*D*) degradation rates decrease, RNA generation rate increases (*E*), stoichiometric factor increases (*F*), and miRNA-target RNA complex dissociation rate decreases (*G*). Parameter units for *b*, *d*_*T*_, *d*_*R*_, *g*, and *u*_*C*_ are s^−1^. The parameter *α* is unitless. Parameter values used are reported in Table S1. To see this figure in color, go online.

Considering the full derivative, as opposed to the partial derivative, accounts for indirect, as well as direct, interactions. This distinction is crucial when considering long-range information flow and not merely nearest-neighbor correlations (33). To analyze *C*(*ℓ*) numerically, we calculated the change in *T*_*ℓ*_ following a small change in *T*_0_. Specifically, we constructed a miRNA-RNA chain with *N* = 100 RNA components. At *t* = 0, the system is in steady state. Then, we decreased the generation rate of T_0_, located in the middle of the chain, so that *T*_0_ → *T*_0_ − Δ*T*_0_, where Δ*T*_0_ ≪ *T*_0_. We then let the system relax to its new steady state, denoting the change in the steady-state level of target RNA *ℓ* by Δ*T*_*ℓ*_. The resulting correlation function is given by *C*(*ℓ*) = (Δ*T*_*ℓ*_/*T*_*ℓ*_)/(Δ*T*_0_/*T*_0_). Here, each parameter for every RNA component was sampled from a suitable Gaussian distribution, located within the biologically relevant range (see Table S1). We repeated the simulations multiple times with different instantiations of the parameters. In Fig. 5 *A*, we present the average over *C*(*ℓ*) values obtained in all simulations. We found that the average *C*(*ℓ*) can be approximated by exp(−*ℓ*/*ℓ*_0_), where *ℓ*_0_ is defined as the correlation length, the distance from the source at which the correlation function decays to 1/*e* of its initial value. For certain parameter samplings, the propagated perturbation effect, as measured by *C*(*ℓ*), might be substantially larger than the average, indicating amplification of the response to a distant perturbation (see also below in the analysis of the propagation of perturbation through a more elaborate network structure).

In addition to the numerical study, we performed an analytical calculation of *C*(*ℓ*) based on the mathematical framework presented in Barzel and Biham (33). Considering the posttranscriptional chain described in Eq. 3, and assuming that the chain has circular boundary conditions, or that 2*N* >> 1, the steady-state solution for the system components becomes

(5) $$\begin{array}{l} T=\frac{1}{4\;\alpha \;\tilde{b}d_{T}}\left\{-2\;\tilde{b}\left(g_{R}-\alpha g_{T}\right)-d_{R}d_{T}+\sqrt{{\left[2\;\tilde{b}\left(g_{R}-\alpha g_{T}\right)+d_{R}d_{T}\right]}^{2}+8\;\alpha \;\tilde{b}g_{T}d_{R}d_{T}}\right\}\\ R=\frac{g_{R}}{d_{R}+2\;\alpha \;\tilde{b}\;T} \end{array}$$

where $\tilde{b}=b\;d_{C}/\left(u_{C}+d_{C}\right)$. We find that the correlation function for target mRNAs, *C*(*ℓ*) = (*dT*_*ℓ*_/*T*_*ℓ*_)/(*dT*_0_/*T*_0_), which depends on the distance (or the number of edges), *ℓ*, between two target mRNAs in the chain, satisfies the equations

(6) $$\begin{array}{l} C\left(0\right)=1\\ C\left(\ell \right)=qp\;\left[C\left(\ell +2\right)+2C\left(\ell \right)+C\left(\ell -2\right)\right],\;\ell \geq 2, \end{array}$$

where $p\equiv -\alpha \;\tilde{b}\;g_{R}/{\left(d_{R}+2\;\alpha \;\tilde{b}\;T\right)}^{2}$, $q\equiv -\tilde{b}\;g_{T}/{\left(d_{T}+2\tilde{b}\;R\right)}^{2}$. Looking for a solution for Eq. 6 of the form *C*(*ℓ*) = exp(−*ℓ*/*ℓ*_0_), we find that $\ell_{0}={\left\{\text{cos}h^{-1}\left[1/\left(2\sqrt{qp}\right)\right]\right\}}^{-1}.$ This solution is valid in the range of parameters for which the condition *qp* < 1/4 is satisfied. A special case that satisfies the above condition is one for which the generation rate of the miRNAs is equal to that of the target RNAs, *g*_*R*_ = *g*_*T*_ ≡ *g*. In this case, defining $X\equiv \tilde{b}\;g/(d_{R}d_{T})$,

(7) $$\frac{1}{\sqrt{qp}}=\frac{1}{2\sqrt{\alpha }\;X}\left[1+2X\left(1+\alpha \right)+\sqrt{{\left(2X\left(1-\alpha \right)+1\right)}^{2}+8\alpha X}\right].$$

**Figure 6:**
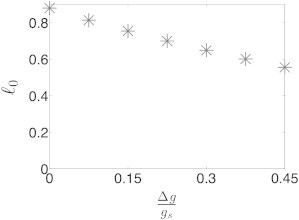
Correlation length versus normalized difference in the generation rates of RNA components. The correlation length increases as the difference between miRNA and RNA generation rates, Δ*g* = *g*_*R*_ − *g*_*T*_, decreases, i.e., as the system approaches equimolar equilibrium of all RNA components. The parameter values used in the simulations are reported in Table S1. Here, we kept *g*_*R*_ constant and set *g*_*T*_ = *g*_*R*_ − Δ*g*.

Overall, we find an analytical solution for the correlation length, *ℓ*_0_ = *f*(*α*, *X*), where it is a monotonically increasing function of *X* and *α*, independently. Therefore, it increases with RNA generation rate and miRNA-target RNA binding rate (as the generation and interaction processes become dominant), and decreases with miRNA and target RNA degradation rates (as the process of degradation becomes dominant). In addition, the correlation length increases with the stoichiometric factor and thus reaches its maximal value for the stoichiometric case, *α* = 1, and exhibits its minimal value for the catalytic case, *α* = 0. Finally, the correlation length decreases with *u*_*C*_, exhibiting its maximal value when no dissociation of the miRNA-target RNA complex occurs (*u*_*C*_ = 0). The analytical result for the correlation length fits well the numerical results, as shown in Fig. 5, *B*–*G*. In addition, we found that the propagation of the perturbation effect along the network strengthens as the system approaches equimolar equilibrium of all RNA components, exemplified by the increase in correlation length as the difference in generation rates between miRNAs and target RNAs, Δ*g* = *g*_*R*_ − *g*_*T*_, decreases (Fig. 6). Thus, as the unperturbed levels approach equilibrium, the correlation length approaches its maximum, meaning that the perturbation effect may propagate and reach farther parts of the miRNA-target RNA network.

**Figure 7:**
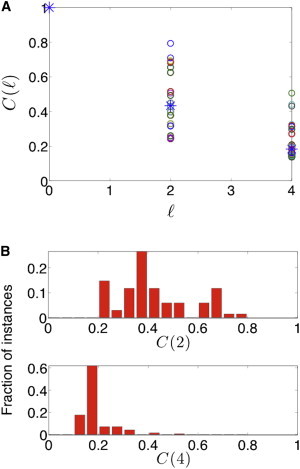
The correlation function for a subnetwork of the human miRNA-mRNA interactome. Shown are the results of the effect of perturbations for the sample of the CLASH-based subnetwork (Fig. 2). Each of the 15 miRNA nodes in that subnetwork was computationally perturbed by decreasing its transcription rate, and the correlation function was computed for all miRNA nodes in the subnetwork. (*A*) Correlation function values for individual miRNA nodes (*circles*) and the average correlation function over all perturbations and all miRNA nodes (*stars*), shown for 2-sep and 4-sep miRNAs. (*B*) Histograms summarizing the correlation function values for 2-sep and 4-sep miRNAs. The parameter values used in the simulations are reported in Table S1. The average correlation function values decrease from the source to 2-sep and 4-sep miRNA nodes. Although these average values may be small (see also Fig. S2), there are individual 2-sep and 4-sep miRNA nodes that are highly affected. To see this figure in color, go online.

Although the chain model illustrates the possible magnitude of the propagation of perturbation effects, it would be very informative to analyze also a dense network, where the same distant ceRNAs may be formed through various paths. Thus, we extended our analysis beyond the linear model to a subnetwork structure based on the experimentally determined human miRNA-mRNA subnetwork presented in Fig. 2 as an illustrative example. This subnetwork includes 15 miRNAs and 6 mRNAs. We performed 15 computational perturbation experiments, where in each experiment, the level of one of the miRNAs (source nodes) was perturbed (its synthesis rate was decreased to 9/10 of its original level), similar to the chain perturbation described above. Note that here, for the purposes of generality, we perturb the miRNA nodes, whereas for the chain analysis we perturbed the target RNA nodes. For each such perturbation, we computed the correlation function for all miRNAs (obtaining a total of 15 × 15 correlation function values). In Fig. 7, we present results for this system, where the generation rates of RNA components were assigned to be proportional to their respective number of neighboring RNA components in the network. In Fig. S2, we present results for the same system, where the generation rates of RNA components were assigned to be equal, resulting in dampened correlation function values for the various miRNA pairs. These analyses demonstrate several aspects of the more complex network structure. Consistent with the results obtained for the linear model, we found that the value of the correlation function (averaged over all pairs of miRNAs in the subnetwork) decreases with the distance from the source, as demonstrated in Figs. 7 *A* and S2. However, many of both the 2-sep and 4-sep miRNA nodes were only slightly affected by the perturbation (as exhibited in Fig. S2), where the effect is diluted due to the subnetwork structure. Still, although the average value of the correlation function may be small, there are specific cases in both the 2-sep and 4-sep nodes where the correlation function value is substantially higher than the average (Figs. 7 and S2). This signifies the notion that although the average effect of cross talk between RNA components in the PTRN may be mild, there could be certain RNAs that would be highly responsive to specific perturbations.

### Proof of concept for cross talk between distant ceRNAs

**Figure 8:**
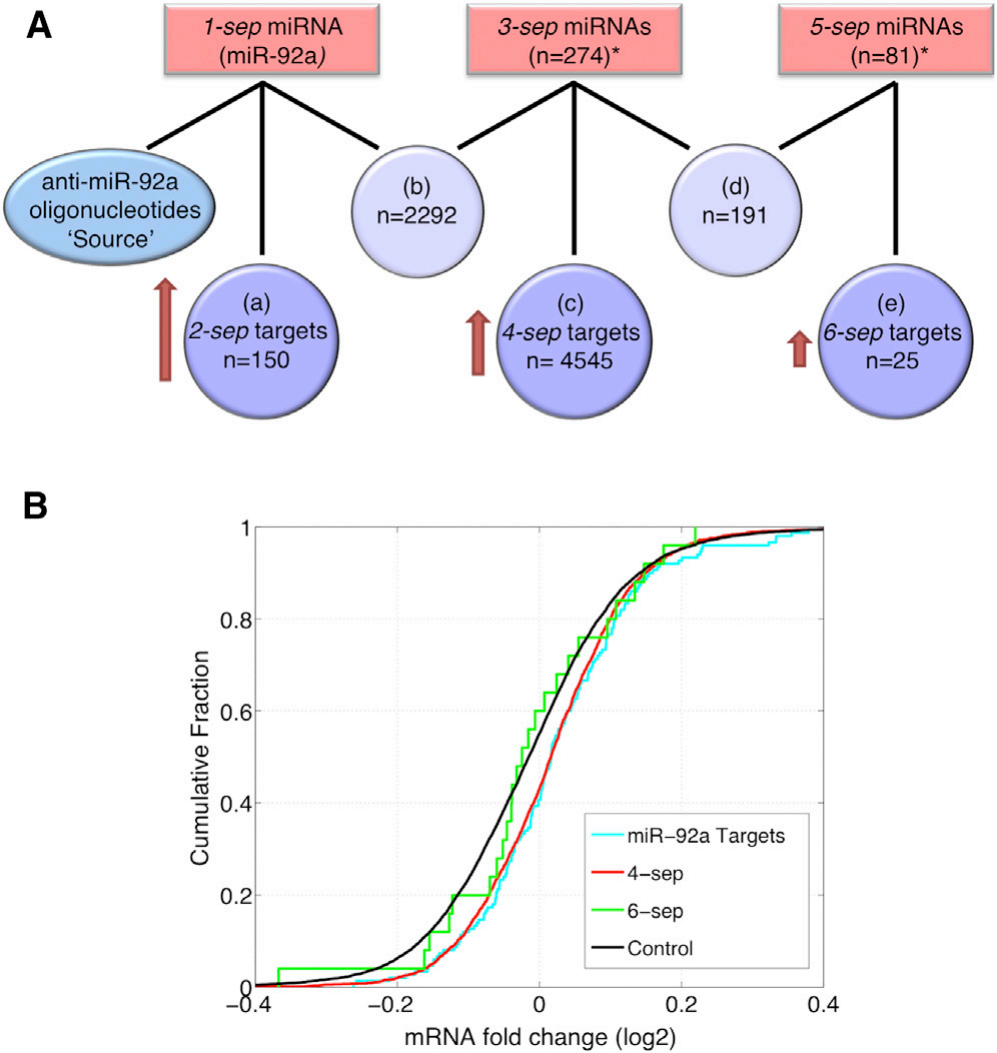
Experimental evidence for distant ceRNAs. (*A*) A representation of the paths originating from oligonucleotide inhibitors of miR-92a along the miRNA-mRNA human interactome based on the CLASH data (25). To follow the perturbation effect, we classified the target groups: targets (2-sep) of miR-92a (1-sep) (*a*); shared targets of miR-92a (1-sep) and 3-sep miRNAs (*b*); targets (4-sep) of the 3-sep miRNAs (*c*); shared targets of 3-sep and 5-sep miRNAs (*d*); and targets (6-sep) of 5-sep miRNAs (*e*). A full list of the miRNAs and targets comprising each of the groups is available in Table S2. In the experiment, miR-92a activity was depleted by the transfection of anti-miR-92a oligonucleotides and the change in expression was measured by microarray. We followed the change in expression of genes in the 2-sep, 4-sep, and 6-sep target groups (*a*, *c*, and *e*, respectively). It was shown that depletion of miR-92a was followed by upregulation of most of its targets (25). The shared targets of miR-92a (1-sep) and 3-sep miRNAs (*b*) are now expected to be more accessible to the 3-sep miRNAs and attract them, weakening the regulation of the 4-sep targets by 3-sep miRNAs, and thus leading to their upregulation. In turn, the shared targets of 3-sep and 5-sep miRNAs (*d*) are expected to be more accessible to the 5-sep miRNAs and attract them, weakening the regulation of the 6-sep targets by 5-sep miRNAs and leading to their upregulation. Numbers of miRNAs given in 3-sep and 5-sep groups (starred in the figure) are for total number of miRNAs that share targets with their previous respective miRNA group. The actual numbers of miRNAs considered as regulators of 4-sep targets and 6-sep targets are smaller (264 and 21, respectively), as many targets were removed from the analysis because they were not uniquely targeted by the respective 3-sep/5-sep miRNAs. (*B*) Cumulative distribution of expression changes after depletion of miR-92a by adding anti-miR-92a oligonucleotides. The log2 fold change of each of the target groups analyzed was compared to a control group of genes not found to be targeted by miRNAs in the CLASH experiment (25). Each of the target groups, the 2-sep targets (miR-92a targets) and the 4-sep targets, were statistically significantly upregulated compared to the control group of nontarget genes (K-S p-values of 5.6 × 10^−4^ and 1.93 × 10^−72^, respectively).

To validate the miRNA-mRNA interactions found by CLASH, Helwak et al. (25) supplemented their interaction data with a microarray expression analysis of HEK293 cells in which miR-92a endogenous activity was depleted. This depletion was achieved by the transfection of miR-92a oligonucleotide inhibitors that compete with its natural targets and thus reduce its endogenous activity. Indeed, Helwak et al. found that miR-92a targets were upregulated in the transfected cells. Thus, the oligonucleotide inhibitor is a ceRNA of the endogenous miR-92a targets. This experiment provided us with suitable data for testing the effects of distant ceRNAs by enabling us to examine the effect of miR-92a oligonucleotide inhibitors (the source) on different RNA components located along pathways originating from it. Gene targets and miRNAs were classified and referred to by their distance from the source node (Fig. 8). miR-92a is termed 1-sep (as it is separated by one edge from the source), and its targets are termed 2-sep. The miRNAs that share targets with miR-92a make up the 3-sep group. 5-sep miRNAs and 4-sep and 6-sep targets were defined according to the same nomenclature (Fig. 8, Table S2). Consistent with the linear model, and to reduce noise, each of the target groups included only targets that are regulated either by a single miRNA (miR-92a (1-sep)) or by multiple miRNAs that belong to the same respective miRNA group (3-sep or 5-sep). In addition, we defined a control group composed of the mRNAs included in the microarray but not targeted by miRNAs according to the CLASH interactome (10,899 mRNAs). According to the premise that there is cross talk between distant ceRNAs, perturbation of the source is expected to lead to perturbation of miR-92a, a weaker perturbation of miR-92a direct target group (2-sep), followed by a weaker perturbation of the 4-sep target group relayed by its 3-sep miRNA regulators, followed by yet weaker perturbation of the 6-sep target group relayed by its 5-sep miRNA regulators. Since miR-92a is depleted, and all miRNAs are negative regulators of their targets, the 4-sep and 6-sep target groups are expected to be upregulated compared to the control group, despite the lack of miR-92a binding sites in their sequences. Analysis of the expression change of the three target groups corroborated this hypothesis (Fig. 8 *B*). miR-92a targets (2-sep) and 4-sep targets showed modest yet statistically significant upregulation compared to the control group (K-S test p-values of 5.6 × 10^−4^ and 1.93 × 10^−72^, respectively). Interestingly, 4-sep targets that are regulated by more than one 3-sep miRNA were found to be statistically significantly more upregulated than 4-sep targets that are regulated by only one 3-sep miRNA (K-S test p-value: 3.38 × 10^−5^), suggesting that the propagated effect is stronger when mediated by more than one miRNA. The 6-sep target group showed no statistically significant upregulation compared to the control group, however, the 6-sep group size is very small and all mRNAs in this group are each regulated by only a single 5-sep miRNA. Expanding the analysis to include low confidence targets (Materials and Methods) led to consistent results for the 2-sep and 4-sep target groups (K-S test p-values: 2.85 × 10^−4^ and 3.38 × 10^−69^, respectively), and a trend of upregulation, although not statistically significant, in the 6-sep target group. Also, the mathematical model predicted that the average change in expression will decrease gradually from miR-92a direct target group (2-sep) to the 4-sep target group and from the 4-sep target group to the 6-sep target group. Although the expected trend is seen in Fig. 8 *B*, the differences between the distributions were not statistically significant. We verified that our results are not biased by indirect transcription regulation interactions and low expression of the control group (see the Supporting Text and Fig. S3). Altogether, our analysis provides a proof of concept for the existence of distant ceRNAs that affect each other's expression through a path of coregulated targets and miRNAs with shared targets.

## Discussion

Accumulating evidence supports a new layer of regulation in the miRNA-target RNA regulatory network mediated by ceRNAs (for review, see Tay et al. (13)). Originally, ceRNAs were defined as target molecules of miRNAs (mRNAs or other RNA molecules) that cross talk via competition over binding to their common miRNA regulators. However, by the same logic, miRNAs that share targets compete over binding to their common targets and therefore also exhibit ceRNA-like behavior, as we and others have demonstrated computationally (16,17,34). Thus, the term ceRNA should be expanded to encompass both types of competing elements. This suggests that perturbations of gene expression can potentially propagate in the network through a cascade of coregulated target RNAs and miRNAs that share targets, leading to mutual effects between distant components in the network, i.e., distant ceRNAs. We demonstrated the possible cross talk between distant ceRNAs using an experimentally determined network of miRNA-mRNA interactions comprised of the recently published CLASH-derived human interactome (25) and characterized both numerically and analytically its magnitude and the parameters affecting it.

A testable prediction of the distant ceRNA hypothesis is that downregulation of a specific miRNA will result not only in the upregulation of its direct targets, but also in the upregulation of distant genes in the network, residing on a posttranscriptional path originating from that miRNA. Indeed, we found that depletion of the hub miR-92a by transfection of the anti-miR-92a oligonucleotides (25) resulted in the upregulation of the 4-sep targets (and to a lesser extent of the 6-sep targets), none of which is a direct target of miR-92a. Furthermore, we demonstrated that the propagated perturbation effect on 4-sep targets is more pronounced for targets that have multiple miRNA regulators in the 3-sep group, further supporting the conclusion that the observed effect is miRNA-dependent. Although these observations support the distant-ceRNA hypothesis, it is still plausible that the upregulation of 4-sep targets could be attributed to other alternative mechanisms. For example, it could be argued that some of the genes in the 4-sep group are targets of transcription factor (TF) activators that are directly regulated by miR-92a whose depletion led to an increase in the TF levels and to a corresponding increase in their target levels. We ruled out this possibility by excluding from the analysis all direct targets of those TF activators and the ceRNAs of those targets, demonstrating that the upregulation of targets in the 4-sep group is still clearly observed. Another plausible explanation for the upregulation of distant ceRNAs regards competition over binding to Argonaute, an auxiliary protein essential for miRNA function. When Argonaute is found in limiting amounts, competition may arise between miRNAs over binding to Argonaute (35), reducing their effectiveness and leading to upregulation of their targets. miR-92a depletion by the transfection of the anti-miR-92a oligonucleotides utilizes cellular Argonaute and thus could reduce its availability. However, as the experiment was performed in HEK293 cells expressing AGO1 in excess, it is conceivable that AGO1 was not a crucial limiting factor. Furthermore, even in the case of competition over binding to Argonaute, the effect of miRNA competition over their shared targets can still be observed (34). Altogether, this suggests that the upregulation of genes along the posttranscriptional path originating from miR-92a is at least partially due to mutual effects between distant ceRNAs.

To characterize the parameters affecting the propagation of perturbation effects through the network we studied it by mathematical modeling and simulation. To keep the mathematical model tractable, we started by analyzing a chain of miRNA-target interactions. The linear model is amenable to analytical treatment, providing exact results that can be used as a benchmark against which experimental and numerical results or more complex networks can be evaluated. The linear model essentially describes the shortest path between each pair of RNAs in the network. In this sense, it is an approximation that neglects the dilution of the signal due to branching of paths as well as its possible enhancement due to reconnections. This approach allowed us to identify and study the main factors affecting the propagation of perturbation effect and to characterize its functional form, as well as capture the general potential for cross talk between distant ceRNAs in the miRNA-target RNA interactome. Interestingly, the average path length of the CLASH-based miRNA-mRNA network is 4, well within the effective propagation distance supported by the model. Our model demonstrates that as the unperturbed levels approach equilibrium, the perturbation effect propagates along longer paths in the network. This finding is consistent with previous studies that reported a miRNA dilution effect, where repression becomes less substantial as the overall concentration of the miRNA targets increases (11,16,17,36,37). Moreover, it concurs with recent theoretical studies reporting that the optimal cross-regulation between coregulated ceRNAs occurs at a near-equimolar equilibrium of all involved RNA components (11,17). Since the fate of the miRNA-target RNA complex is not yet fully deciphered and may vary in different cellular setups between a catalytic and a stoichiometric interaction, the extent of miRNA recycling is not clear (38). Nonetheless, in our model, we show that as long as the regulation has a stoichiometric component, propagation and mutual effects between distant ceRNAs still exist to a certain level, and its magnitude will increase with the extent of the stoichiometric component. This result is in agreement with previous theoretical studies demonstrating that cross talk among coregulated ceRNAs at steady state requires some degree of stoichiometricity (11,16,17).

To further characterize the nature of the cross talk between distant ceRNAs within more realistic and complex networks, we analyzed the mutual effects between RNA components in a subnetwork structure based on the CLASH human miRNA-mRNA network, as depicted in Fig. 2. The highly connected topology of the experimental network results in many nodes being located within a short distance of any source node. On the one hand, this implies that the propagation of perturbation effects should be observed, but on the other hand, the high connectivity may imply possible dilution of the effect of source perturbations. Indeed, we found that, in accord with the linear model, the correlation function values decrease with distance between pairs of RNA components. Although in general the magnitude of distant cross talk may be small, it may be substantial for certain RNA components depending on the features of the network (Fig. 7). Thus, it would appear that distant ceRNA cross talk depends both on the underlying dynamical parameters of the nodes and on the topology of the network.

The modest effects observed for the 4-sep group both by the analysis of the experimental data and by the simulations (Figs. 7, 8, and S2) challenge the biological relevance of remote ceRNA regulation that propagates within the network. On the one hand, it could be an inevitable consequence of the network structure, too subtle to be of any significant biological consequence. On the other hand, certain RNA components may be highly responsive to specific perturbations, depending on the network topology and parameter values (as can be seen from the differences between Figs. 7 and S2), suggesting a possible mechanism for tunable selectivity. Selectivity in this context was also recently discussed for interactions between coregulated ceRNAs, which can be either missing, symmetrical, or asymmetrical (16). Altogether, the average modest effects between distant ceRNAs along with selectivity may guarantee network stability and dampening of perhaps harmful remote effects. Still, in some cases, even small effects caused by remote ceRNAs might be biologically meaningful, as cells may be extremely sensitive to small changes in expression of specific genes, such as in the case of the tumor suppressor PTEN (4,39), highlighting the potential impact of the small effects exerted by distant ceRNAs. Finally, it has been observed often that the effect of miRNAs on the expression level of their targets is quite mild (typically less than twofold) (40). Consequently, the various interpretations as to the functional implications of the mild effects exerted by miRNAs on their targets may be relevant also to distant ceRNAs. These interpretations include fine-tuning of gene expression, global coordinated regulation of a group of genes, complete inhibition of specific key targets, possible buffering of key targets through collective mild regulation of other targets and nonlinear response due to a threshold effect (17,40–48).

The propagated perturbation effect demonstrated here can initiate from any node, either miRNA or target RNA, that is either up- or downregulated, and propagate to any node connected to it in the undirected miRNA-mRNA network. This type of information flow may underlie inexplicable gene perturbation effects. It has been often the case that after gene perturbation (e.g., of TF, miRNA, or drug target), there were genes that changed their expression, whose connection to the perturbed gene could not be traced in the cellular networks. By searching for the ceRNA-mediated connectivity between the perturbed and affected genes, previously unexplained connections between them may be revealed. For example, this hidden layer of connectivity may provide novel insights into different genes and pathways that are impaired in the same genetic disease. Moreover, the ceRNA-mediated information flow may provide an effective mechanism for a fast, yet gradual, transmission of signals, which is programmed into the regulatory networks of the cell. It should be illuminating to revisit the cellular networks and molecular pathways in health and disease while considering the proposed ceRNA-mediated connectivity in the posttranscriptional regulatory networks.

Although the focus of this study was on the miRNA interactome in humans, ceRNA-like effects were reported for other organisms and other posttranscriptional interactions. In particular, bacterial small RNA (sRNA) molecules, which also exert their regulatory function by basepairing with mRNA targets, were shown to be subjected to ceRNA-like regulation as well (44,49,50). One of the advantages of our model is that its qualitative results are insensitive to parameters and thus it can be applied not only to the miRNA interactome but also, for instance, to sRNA-mRNA networks. Indeed, in a model based on repressor sRNA-mRNA interaction parameters, we observed similar propagation of perturbation effects through the bacterial network (Fig. S4). Taking these findings one step further, we would like to argue that other posttranscriptional cellular networks in various organisms may underlie similar mechanisms, provided that the interactions have a stoichiometric component. Appealing candidates would be RNA binding proteins and their targets, where the information flow could be manifested either in changes in mRNA levels or changes in their cellular localization. For each such network, given its topology and specific parameter values, the model can be used for precise prediction of dynamic and steady-state behaviors, e.g., the magnitude of information flow. In addition, controllable experimental setups can be used to verify the dependence of information flow on parameter values. Finally, different posttranscriptional regulators, such as miRNAs and RNA-binding proteins, can potentially join forces in the generation of a ceRNA cascade.

## Supporting Material

Document S1. Table S1, Figs. S1–S4, and Supporting Text

Table S2. Division of high confidence gene targets into three groups based on their position on the pathway relative to the perturbation sorce

Document S2. Article plus Supporting Material
