# Supplementary material for: Interactions between Distant ceRNAs in Regulatory Networks
Source: Biophys J. 2014 May 20;106(10):2254–66. doi: 10.1016/j.bpj.2014.03.040 (PMC4052263; doi:10.1016/j.bpj.2014.03.040)
Supplement: Document S1. Table S1, Figs. S1–S4, and Supporting Text [file mmc1.pdf]

Supporting Material

Interactions between distant ceRNAs in regulatory networks

Mor Nitzan[1,2], Avital Steiman Shimony[2], Yael Altuvia[2], Ofer Biham[1*], and Hanah Margalit[2*]

[1]Racah Institute of Physics, The Hebrew University of Jerusalem, Jerusalem 91904, Israel.

[2]Department of Microbiology and Molecular Genetics, IMRIC, Faculty of Medicine, The Hebrew University of Jerusalem, Jerusalem 91120, Israel.

Table of contents

Table S1 ……………………………………………………………………………2

Figure S1 …………………………………………………………………………3

Figure S2 …………………………………………………………………………4

Figure S3 …………………………………………………………………………5

Figure S4 …………………………………………………………………………6

Supplementary text ………………………………………………………………7

References ………………………………………………………………………9

Table S1

Parameter	Symbol	Units	Default Value			
			Figs. 3, 4 and S2	Fig. 5	Fig. 6	Fig. 7
mRNA transcription rate	g_T	s^{-1}	0.024	2.4e-03 - 2.4e-01 (default 0.024)	1.1e-2 - 2e-2	1e-2-6e-2 *
miRNA transcription rate	g_R	s^{-1}	0.02	2.4e-03 - 2.4e-01 (default 0.024)	2e-2	1e-2-6e-2 *
mRNA degradation rate	d_T	s^{-1}	1e-04	1e-05 - 1e-03 (default 1e-04)	1e-04	1e-04
miRNA degradation rate	d_R	s^{-1}	2.5e-04	2.5e-05 - 2.5e-03 (default 2.5e-04)	2.5e-04	2.5e-04
miRNA-mRNA binding rate	b	s^{-1}	0.001	1e-04 - 1e-02 (default 0.001)	1e-3	1e-3
miRNA-mRNA unbinding rate	u_C	s^{-1}	0.001	0 - 1 (default 0.001)	1e-3	1e-3
miRNA-mRNA degradation rate	d_C	s^{-1}	0.007	7e-3	7e-3	7e-3
Stoichiometric factor	α	1	0.9	0.1 - 1 (default 1)	7e-1	9e-1

The effects of the parameters b, d_T, d_R, g, α and u_C on the correlation function are examined in Fig. 5, B-G in the main text, where each of their values is varied individually.
The parameter values are based on references (1-9).

* The transcription rate of each mRNA and miRNA in the simulations used to produce Fig. 7 was set to 0.01 multiplied by the number of its regulators or targets, respectively.

Figure S1

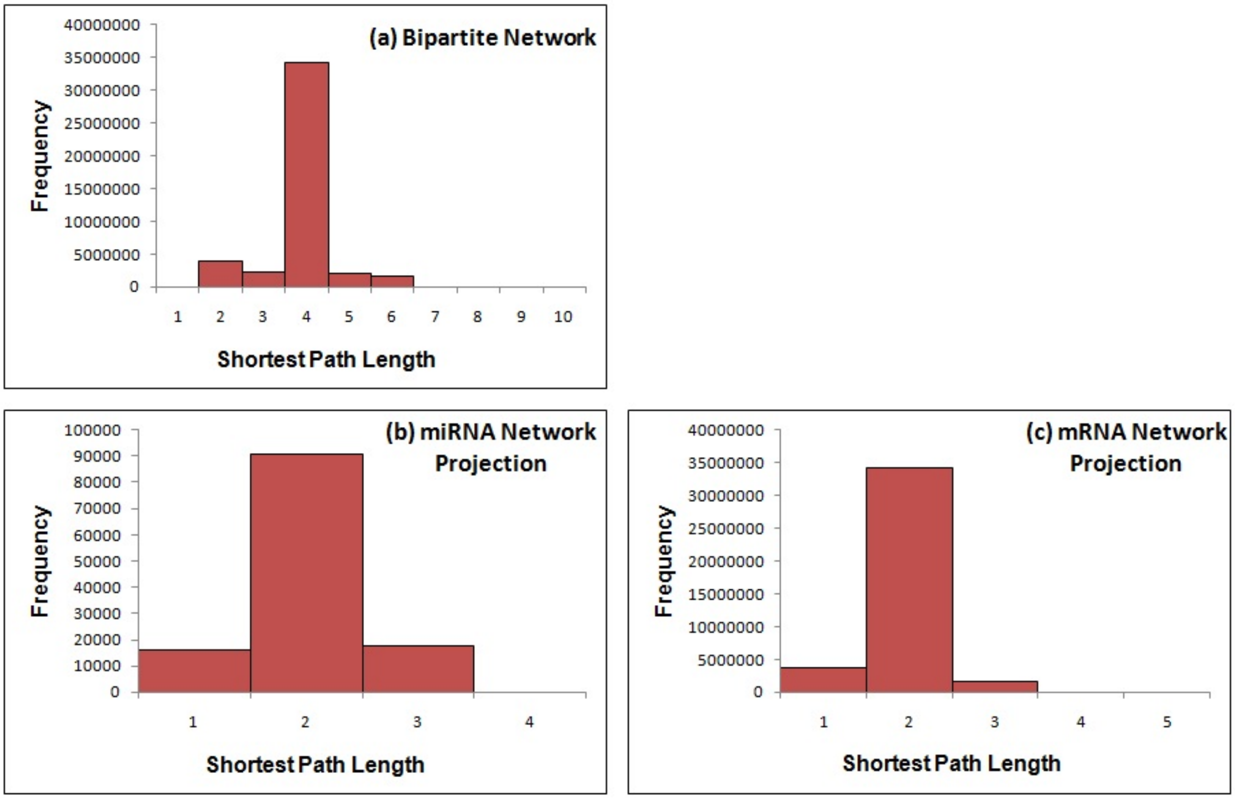

Shortest Path Length Distributions. Presented are the distributions of the shortest path lengths between all node pairs in **(a)** the miRNA-mRNA bipartite network, **(b)** the miRNA-miRNA projected network and **(c)** the mRNA-mRNA projected network. The data is based on the experimentally-determined miRNA-mRNA network by Helwak et al. (10). Note that the y and x axes of the three figures are not drawn to scale.

Figure S2

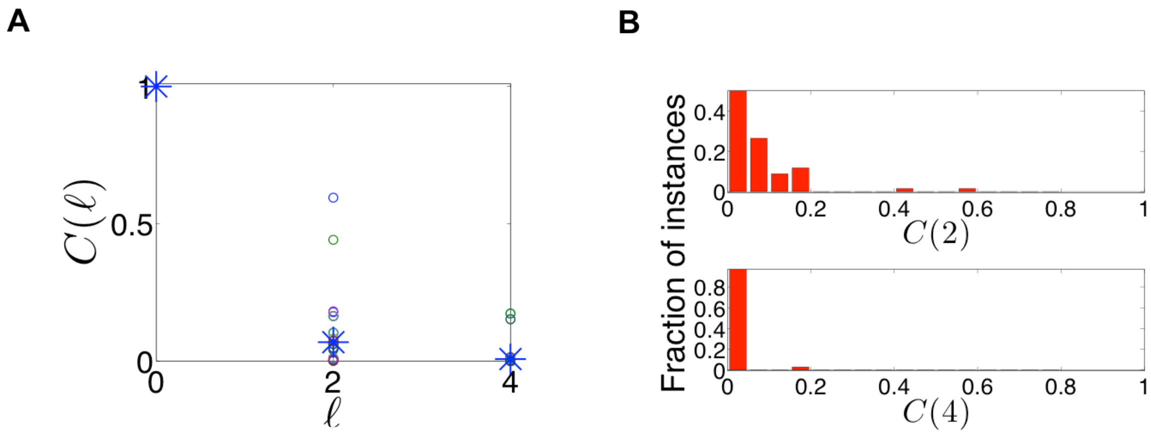

The correlation function for a sub-network of the human miRNA-mRNA interactome. Shown are results of the effect of perturbations for the sample of the CLASH-based sub-network, presented in Fig. 2. Each of the 15 miRNA nodes in that sub-network was computationally perturbed by decreasing its transcription rate, and the correlation function was computed for all other miRNA nodes in the sub-network. *(A)* Correlation function values for individual miRNA nodes (circles) and the average correlation function over all perturbations and all miRNA nodes (stars), shown for 2-sep and 4-sep miRNAs. *(B)* Histograms summarizing the correlation function values for 2-sep and 4-sep miRNAs. While the average correlation function values are small and decrease from the source to 2-sep and 4-sep miRNA nodes, there are individual miRNA pairs among the 2-sep and 4-sep groups that exhibit substantially higher correlation function values. The parameter values used to produce this figure appear in Table S1. Note that, as opposed to Fig. 7 in the main text, the RNA components synthesis rates were assigned to be equal, individually across miRNAs and mRNAs.

Figure S3

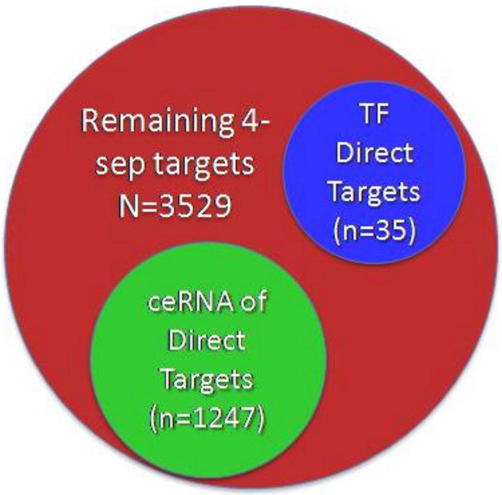

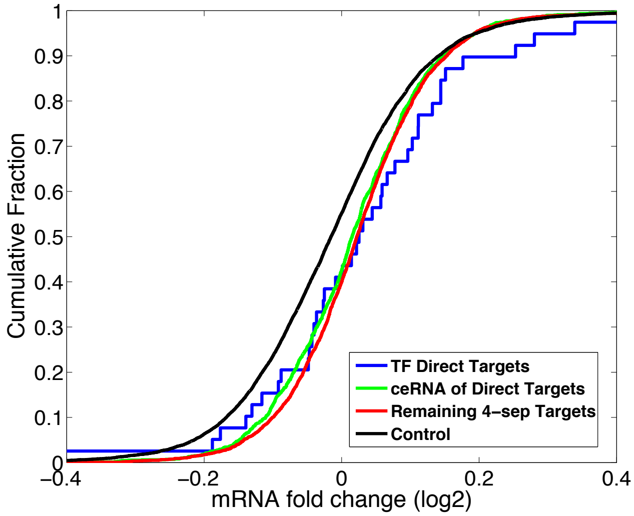

Ruling out possible effects of activator transcription factors (TFs) directly targeted by miR-92a. A) The 4-sep target group (n=4545) was divided into three groups: (i) *TF direct target group*, composed of genes that are direct targets of the 35 activator TFs, which are themselves direct targets of miR-92a. (ii) *ceRNA of Direct Target group*, ceRNA of the targets in (i); i.e., targets that share miRNAs regulators with the TF direct targets. (iii) *Remaining 4-sep target group*, which includes the targets unrelated to the activator TFs targeted by miR-92a. **B)** The log2 fold change of each of the aforementioned target groups was compared to the control group of genes not found to be targeted by miRNAs in the CLASH experiment (10). As expected, the TF direct targets and ceRNAs of direct targets were found to be upregulated (K-S p-value: 0.1198, $1.03x10^{-18}$). Moreover, the remaining 4-sep targets were also upregulated (K-S p-value: $2.04x10^{-64}$), thus demonstrating that the upregulation of the 4-sep targets is independent of this TF effect and can be attributed to the ceRNA-dependent propagation effect.

Figure S4

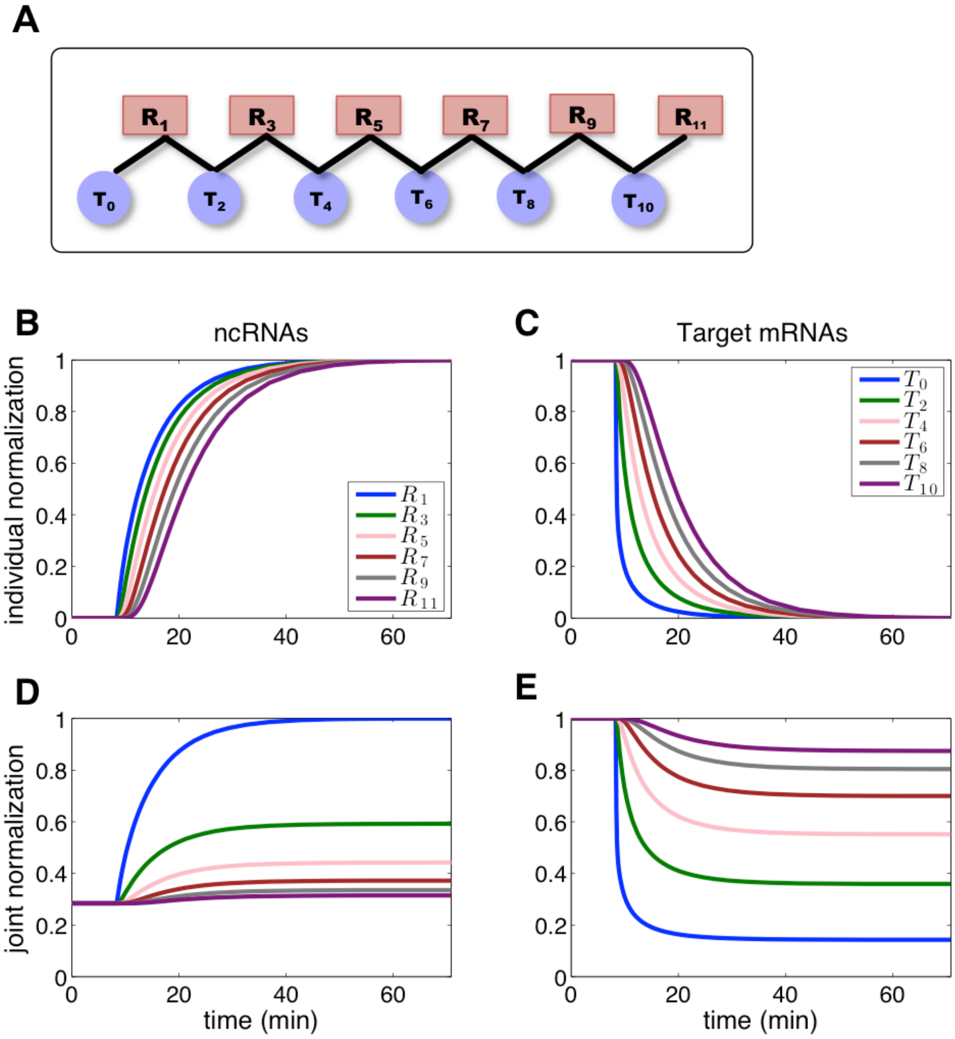

Simulation results of the propagation of a perturbation effect along a chain of interactions between small RNAs (sRNAs) and targets in prokaryotes. In these simulations the sRNAs act as repressors. **A)** Schematic representation of the network comprising recurrent interacting sRNAs and targets, denoted by R_ℓ and T_ℓ, respectively. **B-E)** Perturbation of a sub-network, such as in (**A**), out of steady state by a decrease in the generation rate of one of the targets in the chain, T_0. Shown are the changes in sRNA levels (B,D) and target levels (C, E) over time. (B,C) Levels are normalized by the overall maximal and minimal levels among each individual RNA component. (D,E) Levels are normalized by the maximal level of R_1 (D) or T_0 (E).

A gradual decrease and increase in the levels of mRNA targets and regulator sRNAs, respectively, is clearly observed, consistent with their distance from the perturbed source node, T_0. The parameters used to produce this figure are $b = 0.025\, molecule^{-1} \cdot sec^{-1}$, $g_R = 0.155\, molecule \cdot sec^{-1}$, $d_R = 0.002\, sec^{-1}$, $g_T = 0.15\, molecule \cdot sec^{-1}$ and $d_T = 0.002\, sec^{-1}$. These parameter values lie within a biologically relevant range for bacteria and are based on experimental measurements (11-15).

Supplementary Text

Controlling for gene expression effect

Analysis of the expression levels of the targets that appear in the CLASH data in HEK293 cells according to the Human Protein Atlas (16) revealed that the majority of the targets found by the CLASH method were considered to be expressed at a high or medium level in HEK293 cells. In comparison, the genes from the control group were found to be expressed at lower levels. To negate the possibility that the difference between the fold change levels of the control and the miR-92a targets (2-sep) and 4-sep groups were a result of the relatively low expression in HEK293 cells of the control genes, we re-analyzed that data including only genes whose expression was classified as "high" or "medium" by Danielsson et al. (16). This resulted in new groups such that miR-92a targets (2-sep), 4-sep and 6-sep groups now include 831, 1592 and 4 targets, respectively. The control group now includes 3377 genes. The expression change of the re-classified groups compared to the control showed significant upregulation (K-S p-values: 1.25×10^{-120} and 4.12×10^{-201}, respectively). Thus, the results support that the statistically significant upregulation seen in the 2-sep and 4-sep target groups is not a result of a bias in the endogenous expression levels of the targets.

Controlling for TF effects

In the CLASH data there are direct targets of miR-92a that encode transcription factors (TFs) (35 activators and 24 repressors). Upon depletion of miR-92a activity, their expression level is expected to increase. Consequently the level of the activator TFs' targets and their

corresponding ceRNAs are expected to rise (8). To rule out the possibility that the observed upregulation of the 4-sep targets is due solely to the upregulation of these TF activators, we re-analyzed the data, excluding from the 4-sep target group all genes that are either direct targets of these 35 TF activators (39 genes), or ceRNAs of these TF targets (1247 genes). This left 3529 genes that are not directly affected by the regulation of TF activators that are direct targets of miR-92a (Fig. S2 A). As demonstrated in Fig. S2 B, the upregulation effect is clearly observed for this group compared to the control (K-S p-value: 2.04×10^{-64}), supporting the ceRNA-dependent propagation effect, independent of transcriptional regulation influence. The analysis was not applied to the 6-sep target group as it did not include any targets of TFs regulated by miR-92a. For the analysis of transcription factor effects, classification of activators and repressors as well as their targets were based on the Bioknowledge database (www.biobase-international.com/transcription-factor-binding-sites).

References

1. Hutvagner, G., and P. D. Zamore. 2002. A microRNA in a multiple-turnover RNAi enzyme complex. *Science* 297:2056-2060.
2. Haley, B., and P. D. Zamore. 2004. Kinetic analysis of the RNAi enzyme complex. *Nat. Struct. & Mol. Biol.* 11:599-606.
3. Pillai, R. S. 2005. MicroRNA function: multiple mechanisms for a tiny RNA? *RNA* 11:1753-1761.
4. Kai, Z. S., and A. E. Pasquinelli. 2010. MicroRNA assassins: factors that regulate the disappearance of miRNAs. *Nat. Struct. & Mol. Biol.* 17:5-10.
5. Wang, X., Y. Li, X. Xu, and Y. H. Wang. 2010. Toward a system-level understanding of microRNA pathway via mathematical modeling. *Bio Syst.* 100:31-38.
6. Pasquinelli, A. E. 2010. Molecular biology. Paring miRNAs through pairing. *Science* 328:1494-1495.
7. Baccarini, A., H. Chauhan, T. J. Gardner, A. D. Jayaprakash, R. Sachidanandam, and B. D. Brown. 2011. Kinetic analysis reveals the fate of a microRNA following target regulation in mammalian cells. *Curr. Biol.* 21:369-376.
8. Ala, U., F. A. Karreth, C. Bosia, A. Pagnani, R. Taulli, V. Leopold, Y. Tay, P. Provero, R. Zecchina, and P. P. Pandolfi. 2013. Integrated transcriptional and competitive endogenous RNA networks are cross-regulated in permissive molecular environments. *Proc. Natl. Acad. Sci. U S A* 110:7154-7159.
9. Liu, J., F. V. Rivas, J. Wohlschlegel, J. R. Yates, 3rd, R. Parker, and G. J. Hannon. 2005. A role for the P-body component GW182 in microRNA function. *Nat. Cell Biol.* 7:1261-1266.
10. Helwak, A., G. Kudla, T. Dudnakova, and D. Tollervey. 2013. Mapping the human miRNA interactome by CLASH reveals frequent noncanonical binding. Cell 153:654-665.
11. Milo, R., P. Jorgensen, U. Moran, G. Weber, and M. Springer. 2010. BioNumbers--the database of key numbers in molecular and cell biology. *Nuc. Acids Res.* 38:D750-753.
12. Shimoni, Y., G. Friedlander, G. Hetzroni, G. Niv, S. Altuvia, O. Biham, and H. Margalit. 2007. Regulation of gene expression by small non-coding RNAs: a quantitative view. *Mol. Syst. Biol.* 3:138.

13. Altuvia, S., and E. G. Wagner. 2000. Switching on and off with RNA. *Proc. Natl. Acad. Sci. U S A* 97:9824-9826.

14. Altuvia, S., D. Weinstein-Fischer, A. Zhang, L. Postow, and G. Storz. 1997. A small, stable RNA induced by oxidative stress: role as a pleiotropic regulator and antimutator. *Cell* 90:43-53.

15. Alon, U. 2006. An Introduction to Systems Biology: Design Principles of Biological Circuits. CRC Press.

16. Uhlen, M., P. Oksvold, L. Fagerberg, E. Lundberg, K. Jonasson, M. Forsberg, M. Zwahlen, C. Kampf, K. Wester, S. Hober, H. Wernerus, L. Björling, F. Ponten. 2010. Towards a knowledge-based Human Protein Atlas. *Nat. Biotechnol.* 28:1248-1250.
